# The Challenge of Assessing Mild Neuroinflammation in Severe Mental Disorders

**DOI:** 10.3389/fpsyt.2020.00773

**Published:** 2020-08-20

**Authors:** Karl Bechter

**Affiliations:** Department for Psychiatry and Psychotherapy II, Ulm University, Bezirkskrankenhaus Günzburg, Günzburg, Germany

**Keywords:** neuroinflammation, mild encephalitis, autoimmune encephalitis, autoimmune psychosis, Borna virus, psychoimmunology, parainflammation, neuroprogression

## Abstract

Recent psychoneuroimmunology research has provided new insight into the etiology and pathogenesis of severe mental disorders (SMDs). The mild encephalitis (ME) hypothesis was developed with the example of human Borna disease virus infection years ago and proposed, that a subgroup SMD patients, mainly from the broad schizophrenic and affective spectrum, could suffer from mild neuroinflammation, which remained undetected because hard to diagnose with available diagnostic methods. Recently, in neurology an emerging new subgroup of autoimmune encephalitis (AE) cases suffering from various neurological syndromes was described in context with the discovery of an emerging list of Central Nervous System (CNS) autoantibodies. Similarly in psychiatry, consensus criteria of autoimmune psychosis (AP) were developed for patients presenting with CNS autoantibodies together with isolated psychiatric symptoms and paraclinical findings of (mild) neuroinflammation, which in fact match also the previously proposed ME criteria. Nevertheless, identifying mild neuroinflammation *in vivo* in the individual SMD case remains still a major clinical challenge and the possibility that further cases of ME remain still under diagnosed appears an plausible possibility. In this paper a critical review of recent developments and remaining challenges in the research and clinical diagnosis of mild neuroinflammation in SMDs and in general and in transdisciplinary perspective to psycho-neuro-immunology and neuropsychiatry is given. Present nosological classifications of neuroinflammatory disorders are reconsidered with regard to findings from experimental and clinical research. A refined grading list of clinical states including “classical” encephalitis, AE, AP/ME,and newly proposed terms like parainflammation, stress-induced parainflammation and neuroprogression, and their respective relation to neurodegeneration is presented, which may be useful for further research on the possible causative role of mild neuroinflammation in SMDs. Beyond, an etiology-focused subclassification of ME subtypes, like autoimmune ME or infectious ME, appears to be required for differential diagnosis and individualized treatment. The present status of the clinical diagnosis of mild neuroinflammatory mechanisms involved in SMDs is outlined with the example of actual diagnosis and therapy in AP. Ideas for future research to unravel the contribution of mild neuroinflammation in the causality of SMDs and the difficulties expected to come to novel immune modulatory, anti-infectious or anti-inflammatory therapeutic principles in the sense of precision medicine are discussed.

## Introduction

The field of psychoneuroimmunology has rapidly evolved from early findings of minor systemic alterations indicating the potential involvement of inflammation in various severe mental disorders (SMDs) to a plethora of robust findings demonstrating the rather definitive role of systemic inflammation in a relevant subgroup of SMDs (1–5). However, the exact roles of inflammation and neuroinflammation (whether contributory or causal) in the immune-inflammatory pathophysiology of SMDs remain unclear and even controversial. While SMDs associate with both findings of overactivation and downregulation of the immune system respectively inflammation and anti-inflammation, it appears to be a known phenomenon from autoimmune disorders or cancer, such can be understood as disturbed balance in the process of inflammatory responses and immune regulatory players involved, with a balancing or pleiotropic role to come back to homeostasis, as evidenced for specific cells or signaling molecules (6–8). Given such ambiguity and pleiotropy of single factors found aberrant and representing risk factors of disease, it is not surprising, that there seems little direct (in time periods of actual diseased states) correlation in between these known risk factors and changing clinical pictures in the individual case (at least on the known level of systemic inflammation and/or immune dysregulation), which may heavily depend from environmental factors, eg. infections and stress (9–11). Relevant clinical syndromes included those on the affective to schizophrenic spectrum (not reviewed here), which is unsurprising with regard to often voiced issue of “non-specificity” in psychiatric research as a general problem and relates to the new approach with Research Domain Criteria (RDoCs) in psychiatric research [see (12)].

Not least, in psychoimmunology/immunopsychiatry it remained unclear whether the found systemic immune-inflammatory aberrancies may represent a relevant pathological process in itself potentially involving secondarily the central nervous system (CNS) or whether these systemic aberrancies represented a sign of neuroinflammation manifested (a little) also in the systemic periphery, an important difference. Post-mortem findings rather suggest that indeed low-grade neuroinflammation may be present in a subset of patients in schizophrenia (13, 14): specifically, about 20-40% of brains exhibited signs of inflammation in the frontal lobe, an increased number of macrophages, or an increased number of T lymphocytes and cytokines (14–16); these findings could support the mild encephalitis (ME) hypothesis.

The ME hypothesis was developed in context with research on the possible role of the highly neurotropic Borna disease virus, a known cause of meningoencephalitis in animals, for human mental disorders (17). The ME model was generalized beyond BDV infections, because similar pathogenic mechanism of CNS infection and infection-triggered autoimmunity appeared potentially relevant with many other infectious agents or the knowledge in systemic autoimmune diseases: there are typically many known contributing factors for disease, such as agents, genes, immunity, trauma, toxicity and environment. Also known general epidemiological and clinical/paraclinical data would match with ME hypothesis, even with a considerable subgroup of ME cases in SMD cohorts (18).

Unfortunately, the optimal procedure to diagnose neuroinflammation (to be differentiated from systemic inflammation) *in vivo* in patients with schizophrenia or other SMDs remains undefined, although an increasing number of findings suggest a contributive or causal role of neuroinflammation in SMD subgroups (14–29). The recent discovery of an emerging number of CNS autoantibodies and a greater understanding of autoimmune encephalitis (AE) in neurology (19) has helped a lot to diagnose previously undiagnosed psychiatric cases of AE and to establish a new subgroup of autoimmune psychosis (AP), involving SMD cases presenting CNS autoantibodies with predominantly psychiatric manifestations diagnosed by international consensus criteria, which appear conservative designed and close to the international AE consensus criteria (20, 21). It might be noted, that AP cases and even some AE cases match also the previous ME criteria [compare (20, 22, 23)]. In addition, an increasing number of single case reports about possible cases of AP/ME was made plausible to prevail without presenting (at least known) CNS antibodies but with proven neuroinflammation by brain biopsy, or with new CNS autoantibodies, or cases of subtle epilepsy diagnosed by refined differential diagnosis by EEG, number of the possible AP cases being responsive to immune-modulatory treatments (24–31). Apparently, these recent developments in diagnosis and differential diagnosis of SMDs as APs or possible APs match with the ME hypothesis, which appears well supported when cases demonstrate positive responses on immune modulatory treatments comparable to the treatments used with AE.

This recent development suggested to focus here on the many remaining challenges to assess mild neuroinflammation in SMDs and respective limitations of present diagnostic methods. An improved and timely *in vivo* diagnosis of mild neuroinflammation in SMD patients may consequently lead to improved etiology-guided immunomodulatory, anti-infectious and/or anti-inflammatory treatments. Especially several immune modulatory treatments were rather successful recently, working even in short time, though involving only a small but emerging subgroup of SMD cases, which are now diagnosed as AP or ME, before severely or widely therapy resistant to established treatments. Another difficult question herein appears, to develop further criteria for a possible clinical relevance of mild neuroinflammation/immune dysregulation, when assessed in the individual SMD case by various clinical methods.

Accordingly, the idea of this paper is: 1.To discuss unsolved aspects of clinical terminology and a path to clinically useful classification of mild neuroinflammation, embedded in a wider graded nosological framework from classical encephalitis and autoimmune encephalitis to milder forms of neuroinflammation including ME, parainflammation and neuroprogression. 2. To list an etiology-focused scheme of ME subtypes with respect to available (including preliminary) evidence about the respective roles and interaction between infections and autoimmunity and other contributing factors including genes and environment. 3. To present an overview about the diagnostic and therapeutic approach to mild neuroinflammation by the most actual example AP and respective diagnostic limitations and challenges. 4. To consider new ideas for future research with respect to many open questions about the possible contributive or causal relevance of mild neuroinflammation in SMDs.

## Infections, Autoimmunity, and Possible Me—Human Borna Disease Virus Infection as an Unsolved Example And Beyond

### Human Borna Virus Infection as a Model

When proposing ME hypothesis, ME was assumed to prevail as unrecognized resp. hard to detect low-grade neuroinflammation present in certain brain regions only (therefore “mild” encephalitis as compared to “classical” encephalitis), that was suspected to causally underlie the observed psychopathological syndrome in a subgroup of patients with SMDs, thought to be most relevant in patient cohorts of the (broad) affective and schizophrenic spectrum. This hypothesis was developed during research on the possible role of the highly neurotropic Borna Disease Virus (BDV) in neurological and psychiatric disorders of unknown origin [reviews in (17, 18, 32)]. In short: A small subgroup of neurological (about 4,5%) and psychiatric patients (about 6%) presented antibodies against BDV, suggesting a possible causal involvement of BDV, because the seroprevalence was increased compared to normal controls (about 3,5%) in our region, endemic for BD. In addition, BDV seropositive neurological patients had an increased prevalence of cases hospitalized because of lymphocytic meningoencephalitis and BDV seropositive psychiatric patients presented with an increase of small brain lesions in MRI (33) and/or minor cerebrospinal fluid (CSF) abnormalities indicating an intrathecal immune response (34). These findings suggested the possibility that both these clinical syndromes, neurological syndromes of lymphocytic meningencephalitis and psychiatric syndromes (SMDs including various types of broadly defined affective and schizophrenic spectrum psychoses) were caused by an underlying infection by BDV, in some cases with neurological and psychiatric syndromes even clustering in families (35). From experimental research, two different pathomechanisms underlying the clinical syndromes appeared plausible mechanisms (18, 36–39): acute mild localized (preferential brain region involved was the limbic system) infectious encephalitis or autoimmunity triggered by the BDV infection (40), and thus both pathomechanisms might in principle have the potential to induce a spectrum of psychiatric syndromes depending from a variety of known contributive factors including immune status of the infected, age, genes and others [compare (18, 36)]. However, non-deadly but clinically relevant brain infection or CNS autoimmunity or ME was difficult to prove *in vivo*, not least from ethical reasons.

Interestingly, acute (classical) infectious BDV meningo-encephalitis was only recently proven in humans, in three patients having died from the disease, only 1 case from spontaneous natural infection the other 2 cases after organ transplant (41, 42). The responsible BD virus was renamed BoDV-1 after the discovery of several BDV variants some years ago. Beyond, in mainly retrospective analysis of brain material (+ CSF+ Sera) from cases having died from undefined lymphocytic encephalitis, further eight cases of BoDV-1 encephalitis were confirmed very recently (43). Importantly, the conclusions of Niller et al. in that “The possibility of mild, asymptomatic, or oligosymptomatic courses of BoDV-1 infection cannot be excluded and requires further investigation.” match well with our previous perspective, including the point that antibody and immunoglobulin detection in CSF are most relevant for diagnosis. In sum, the difficulties of *in vivo* diagnosis of milder forms of BoDV-1 infections are apparent. In this context, one should not forget that diagnosis can be difficult even in some cases of classical encephalitis, although the field is equipped with established diagnostic methods [compare (44, 45)] and one should recognize, that the neuroinflammatory process in “classical” (meningo)-encephaltis is rather severe and distributed as compared to the proposed ME concept, and this apparently associates with important differences for the sensitivity and respective limitations of diagnostic methods.

Antiviral therapy in BoDV-1 encephalitis was and is still until now unavailable [compare (43)]. Given there were two alternative types, infectious ME or infection-triggered autoimmune ME, to be considered as underlying pathology in SMDs in BDV seropositive patients, the best option to follow in research appeared to us represented by an autoimmune ME model in BDV seropositive psychiatric patients: such model appeared to match with delayed (months) therapy resistant disease courses, which prevailed in a majority of hospitalized BDV seropositive patients, whereas an infectious ME model might match with short disease courses (days or weeks). This evaluation was a starting point for treatment trials with CSF filtration performed over some years around 2000 in BDV seropositive psychiatric patients, when resistant to established treatments, although admittedly the autoimmune ME hypothesis was speculative. Around that time, CSF filtration had been successfully used to treat therapy resistant cases of Guillain-Barre–Syndrome (GBS) [see results of a randomized trial in (46)]. GBS is typically triggered by various infections, and such could represent an analogy of the suspected autoimmune ME triggered by BDV. Thus, an experimental trial with CSF filtration in selected SMD patients appeared acceptable and was continued later in trial approved by ethical committee (supported by funding from Theodore and Vada Stanley Foundation). Indeed, CSF filtration series over usually five days, similar to CSF filtration trials in GBS, were performed as add-on treatment to unchanged established psychopharmacological treatment and led to significant improvement or remission in about two-thirds of BDV seropositive psychiatric patients (n =10, not included repeat filtration series); interestingly, CSF filtration was effective typically in short time (days) in these patients who had been therapy-resistant for many months before (47–50). While some of these patients relapsed, repeated CSF filtration led to remission again, and some patients were stable over years after CSF filtration treatment. These successful CSF filtration treatment trials indirectly supported the previous model of autoimmune ME, because of its apparent immune modulatory mechanism of action shown in GBS. Of note, the various antineuronal antibodies known now, were not discovered at that time [see also the recent debate about the definitions of AP and AE in Lancet Psychiatry (20)]. Experimental CSF filtration was intriguingly successful in a small subgroup of BDV seropositive cases, but was not further followed from several reasons, not least because being technically rather challenging.

### Generalizing the ME Model

The ME hypothesis claimed, that it was difficult to clinically assess mild neuroinflammation in patients even during clinically relevant stages of disease, regardless of the underlying cause of neuroinflammation and/or contributing pathomechanisms [see (17)]. Thus, the assessment of mild neuroinflammation and immune-pathological mechanisms involved needed to be elucidated in the first place, to then select potential therapy options based on the available evidence of contributing pathomechanisms. For example, given autoimmune ME can be triggered by various infectious agents or by an unknown endogneous immune system related causality, preferential therapeutic goals might be to search for normalization of an exaggerated immune response, or alternatively to halt the triggering mechanism. The various immune modulatory treatments now used in AE (51), similarly recommended in AP (21), seem to follow such principle, ie. searching for CNS autoantibodies and evidence of neuroinflammation in the individual case.

It is now accepted, that CNS specific autoantibodies play an important pathogenic role in AE (52, 53) and probably in AP (21). Nevertheless, strictly speaking, proof of AE as an autoimmune disease is still missing in that only one of four analogous Koch`s postulates was fulfilled (54). In addition, the trigger of the autoimmune dysregulation presenting as AE or AP is mostly unclear, if not an underlying tumor was detected (19, 55) or specific infections, like Herpes virus encephalitis, was preceding (56). Nevertheless, although “the discovery of antibodies targeting synaptic proteins has completely changed the approach to neurologic and psychiatric disorders that were previously considered idiopathic or not immune mediated” (57), still many open issues in antibody-mediated encephalitis prevail (58). In addition, hardly anybody might doubt the validity and clinical importance of the AE or the AP concept, which helped with the newly introduced immune modulatory treatments so many severely diseased neurological patients and a still small but emerging number of therapy resistant SMD patients, in the latter group raising a number of difficult ethical questions (59).

Thus, a historical perspective may be of notice here: when critically checked, there is still up to now no definite scientific proof (in the sense of Koch`s postulates) of the causality of spirochetal infection for the delayed tertiary stage of syphilis, as in only very few brains of people died from general paresis the infectious agent was demonstrated (60). Nevertheless, the accumulated findings from epidemiology and clinical research and, not least if not most important, the positive therapeutic response of GP cases to antibiotic treatment (Note: in the early years from about 1907 until to the introduction of penicillin the best established treatment was artificial Malaria infection) clearly support the causality of spirochetal infection for GP. The ethical issues involved in such limited proof of causality in humans may similarly limit the quality of proof achievable in AE, AP, ME and the proposed lower grades of neuroinflammation suspected to causally underlying or contributing to SMDs.

In the framework of ME hypothesis many contributing factors beyond infections, which may play a primary triggering role, are to be considered, especially genes related to inflammatory response system and immunity, actual status of the immune system at time of infection, age of the infected, endogenous factors related to genes like toxicity, various and variant environmental factors like stress, and even chance [compare (18, 61)]. And such or rather similar scenarios are often voiced in psychiatric research now (1, 3, 62–65). Given such perspective is relevant for research on the causes of SMDs, a most important issue represented to assess the level or grade of neuroinflammation and/or immune dysregulation in the individual patient in the respective diseased state, to be defined by consensus criteria and applicable in clinical reality with respect to limitations and availability of diagnostic methods. I think, that in such scenario ME, beyond other grades of mild neuroinflammation, should represent an issue of foremost interest, because ME would represent a grade of neuroinflammation close to classical encephalitis and thus a state of likely clinical relevance and chance for valid assessment in clinical reality plus chance for targeted therapy when ME was identified (see paragraph 3 for more details continued).

Another very special issue with regard to a generalized model of ME hypothesis may represent autoimmunity associated with persistent infections, which can have apparent clinical relevance for SMDs according to recent knowledge, accumulated mainly in children and adolescents. For example, pediatric autoimmune neuropsychiatric disorder associated with streptococcal infections (PANDAS) was related to streptococcal-triggered autoimmunity and according to recent findings even extended to variety of infections and renamed pediatric acute-onset neuropsychiatric syndrome (PANS) (66). Emerging research demonstrated, that PANS may require rather complex treatments that target both the infectious agent and the autoimmune process triggered by the primary infection (67). Reports about CNS related autoimmunity with persistent infections associated with SMDs are very rare in adults [eg (68)], but one should not exclude that such may relate to a gap of knowledge. An elusive review about these issues and principles involved was presented by Platt et al. recently (69): these included as main message to critically observe and recognize the variety of access routes of antibodies and cells to the CNS, beyond the regionality of the blood-brain-barrier (BBB) and the blood-CSF-barrier (BCSFB), and as far as possible, to assess respective barrier alterations in the individual case, and to identify the possible triggers of autoimmunity in AE or PANDAS, and to consider these triggers for respective targeted treatments, which may need to probe anti-infectious treatments even in the first line. Also this review clearly shows, that eventually a more detailed classification including the refined assessment of BBB and/or BCSFB disturbances, and not least the assessment of latent infections in the diseased individual would be required for optimal diagnosis and treatment of such SMDs, when recognized as AE or AP or ME or maybe of lower graded categories of mild neuroinflammation. One should not forget here also the limited knowledge about rare latent or unusual infectious agents, which may also play some yet undefined role, for example Bartonella species (70) (not to repeat BDV here, see above).

One step forward in this scenario of open questions was in my opinion, to adopt a refined graded consensus classification of clinically relevant neuroinflammation. Neuroinflammation has to be clearly differentiated from systemic inflammation, both in language and clinical approach, although there exists relevant interaction in between periphery and CNS [compare (71)], because the careful assessment of grades of neuroinflammation in diseased states would represent a precondition to inform about a possible clinical relevance of mild neuroinflammation. I therefore outline a respective proposal on refined graded categories of neuroinflammatory disorders according to available pathophysiological evidence in the next paragraph.

## Refined Grading and Nosology of Mild Neuroinflammatory Disorders

The idea that refined grading of neuroinflammation appears clinically relevant was followed by number of authors recently in basic immunology and psychoimmunology research and clinical field (see above and below). Based on broad experimental knowledge, the term parainflammation was proposed for refined grading of inflammation in general (72). In psychoimmunology research field the term was adopted and refocused on the CNS respectively neuroinflammation, but restricted to stress-induced parainflammation (73). Stress research is an interesting, relevant and predominant field in experimental psychoimmunology, but other triggers than stress, eg. infections or immune dysregulation, should not be disregarded as primary triggers for CNS specific parainflammation. Clinical findings with experimental evidence helped to conceptualize neuroprogression in SMDs (65, 74). Neuroprogression appears closely related to neurodegeneration (75, 76), but an exact differentiation was difficult (77).

These new concepts about low grade neuroinflammatory categories/states, including ME, have difficulties to exactly and reliably define and assess the respective state in clinical approach, less so in experimental approaches on which the clinical field can thus build up. Therefore, these new developments appear appropriate and important for further research and may over the long-term prove clinically useful. Some of the difficulties of defining the new concepts clearly relate to the *a priori* difficulties when translating knowledge from experimental field into the clinic from many reasons, especially ethical reasons. However, important and hard to understand in a categorical schematic of pathology (or not) appears the ambiguity of functional alterations itself, in other words an often ambiguous role of single factors in pathogenesis can not rarely nor be categorized as advantageous versus disadvantageous nor pro- versus anti-inflammatory. For example: Neuroinflammation can be differentiated from neurodegeneration by cytokines panels but may require under others to identifying the respective cellular sources of cytokine production which can be assessed in experimental approaches, however for a generalized interpretation of cytokines aberrancies it is hard to come to valid conclusion, as in principle such cytokines increases or decreases of specific cytokines may especially in intermediate states of neuroinflammation have unclear or contrarious meaning depending from different *a priori* settings of the network or context of action (78). Or: The heterogeneous population of myeloid-derived suppressor cells plays an important role in chronic and adaptive immune regulation in cancer and autoimmune disorders, signaling by exosomes appears herein very important in short- and long-distance action, but the final outcome of their respective action appears janus-faced (8): again the resulting pathogenic or non-pathogenic effect of the cellular alterations depend from the state of networks and players involved. Therefore, for clinical decision making the assessment of such type of inflammatory and immune signaling or cell states is not the singular problem, instead also the appropriate classification of effect in a broader picture of interactive players involved. The research on the signaling role of exosomes in CSF represents such a new area of interest in neurology and psychiatry, but is just beginning (79–82). An intriguing yet widely neglected immune regulatory part herein may be played by the exosomes and cells passaging along cranial and peripheral nerves with CSF outflow into peripheral tissues, where CNS specific immune responses are generated then (11, 83–85), to be considered with ME and mild neuroinflammation in future research. Another obvious example for such ambiguity of findings in SMDs are the confirmed immune and neuroinflammatory aberrations in bipolar disorders (11, 86–89): the respective interpretation of immune cell aberrations in blood may make sense only in context with the role of changing environment and possibly the immune response to infectious agents and other factors involved, as these immune aberrancies do not simply correlate with the diseased state of bipolar depression or mania specifically, rather are changing over time in contrarious way (9, 10), which might be interpreted as a slight primary immune defect followed by partially exaggerated and later exhausted immunity (9). Such examples of apparent open research difficulties could be continued, but the aim of this paper is not to provide a comprehensive review about these complicated issues.

I intend to propose and discuss a panel of clinical relevant classifications of mild neuroinflammatory states for use in further research. This proposal will need verification and surely emerging better definitions eventually by consensus. I realize many *a priori* weaknesses because of a limited overall knowledge base. Such limitation must not disregard such undertaken as potentially useful, given that even established clinical terms in neurology and neuropsychiatry related to “classical” neuroinflammation, appear to be more weakly defined, when critically considered in detail, as many clinicians may assume (90): for example, only consensus case definitions are available for such clinically important term like encephalitis: respective clinical assessment was evaluated difficult in some cases, the “classical” definition mainly derived from infectious encephalitis (44). The new term AE appears even more limited, also based on consensus case definition (see above). It is unsurprising on the other hand, that newly proposed terms classifying milder grades of neuroinflammation, such as ME, parainflammation, neuroprogression can only preliminarily be defined for clinical use and it appears justified, that the clinical field is considerably hesitating to adopt such terms. Nevertheless, even the best clinically established diagnosis of “classical” encephalitis appears to be rather imprecise in very detail although valid by consensus case definitions, a generalized theoretically sound definition of encephalitis for interdisciplinary use being not available (90). The imprecision of clinical encephalitis diagnosis is further highlighted when just going into detail of the newly diagnosed cases of BoDV-1 encephalitis by post mortem diagnosis [see above and compare (43)]: in post mortem examination some of the cases finally classified as BoDV-1 encephalitis showed panencephalitis, or meningoencephalitis, possibly accompanied by hypohysitis or myelitis, not to speak about the found varying distribution of virus within the CNS. In contrast, the clinical diagnosis on time of death was simply “lymphocytic meningoencephalitis” in all cases.

The limited precision and nearly unnoticed change of meaning of clinical terms over time should in addition be recognized, being apparent also in the recent short history of AE diagnosis, thus an important example for our discussion as being closely related to AP [compare also (91)]: from its initial description of AE cases suffering from “classical” limbic encephalitis as paraneoplastic disease, AE has broadened its meaning to now including a larger and seemingly emerging subgroup of cases presenting with minor neurological symptoms, associated with paraclinical findings of neuroinflammation (92, 93), and even with predominant psychiatric symptoms in initial stage with the new consensus criteria (51), in other words now AE representing a milder form of encephalitis previously not diagnosed as encephalitis in neurology, representing an important clinically relevant change (58). Apparently, the discovery of NMDAR antibodies (94) and a still emerging number of newly discovered CNS autoantibodies (23, 92, 95–97) was a major criterion to newly diagnosing many cases of AE that were not diagnosed as encephalitis before, and this includes a subgroup of SMDs diagnosed as AE (20, 22, 98), recently enriched by cases with pure psychiatric syndromes termed AP (20). However, one should recognize, that CNS autoantibodies (in blood) may prevail also in considerable part of normal controls (99–102), raising difficult questions and controversy about the pathogenicity of these antibodies (52, 99, 101–103).

One might remember here again the long-standing highly controversial sights about neuroinflammation versus neurodegeneration and classification of late stage syphilis, a disease of eminent relevance in psychiatry at the time around 1900: too strictly separating post-mortem findings of neuroinflammation versus neurodegeneration was a major obstacle for understanding the common bacterial etiology of general paresis presenting with two main but differing types of CNS pathology (60).

In sum, I feel prepared to present here a proposal of refined clinical differential diagnosis of mild neuroinflammatory disorders, which, though preliminary with regard to criteria-based definition, might turn out to be helpful for further research onto the causality of mild neuroinflammatory states in a subgroup of SMDs. Such attempt would match with the goal of precision psychiatry [compare (63)]. I present this list with an overview and hierarchical range from classical neuroinflammatory disorders to suspected/proposed refined graded subtypes of neuroinflammation (**Table 1**) and further an etiology-differentiated sub-classification of various MEs (**Box 1**).

**Table 1:** Clinically established and newly proposed terms to classify neuroinflammation ranging from “classical” definition of encephalitis to “milder” forms such as AE, AP, ME, parainflammation and neuroprogression, to neurodegeneration (References see text). Abbreviation: CNS, central nervous system.

Category	Nosological Principle	Status/Advantage	Limitations
**Meningoencephalitis**	generalized inflammation of meninges and CNS	clinical consensus case definition, established	meningitis diagnosed by clinical examination
**Encephalitis**	generalized or definite localized CNS inflammation	clinical consensus case definition, established	sensitivity/validity of diagnostic methods
**Autoimmune Encephalitis (AE)**	localized, rarely generalized, autoimmune-related CNS inflammation or suggested by surrogate consensus markers	clinical consensus case definition, including cases not diagnosed by classical criteria of encephalitis	diagnostic methods; symptoms when classical ones missing; consensus therapy emerging
**Autoimmune Dementia, Autoimmune Epilepsy, Autoimmune Movement Disorder**	neurological syndromes with isolated or prominent clinical features recognized as of autoimmune etiology; subtypes of AE	emerging clinical consensus, specifically addressing previously unknown etiology and (immune) therapy	diagnostic methods predominant for diagnosis; therapy emerging
**Autoimmune Psychosis (AP)**	psychosis recognized as of autoimmune etiology, similar to isolated neurological syndromes; additional cases with novel, currently unknown CNS antibodies suspected; subtype of AE	first international consensus on conservative case definition with (known) CSF antibodies; successful treatment approaches emerging	diagnostic methods predominant and emerging; therapy emerging
**Mild Encephalitis (ME)**	suspected localized low-grade neuroinflammation causing severe mental illness	emerging support for previously unknown prevailing autoimmune/infectious ME	a priori difficult to prove as clinical entity
**Parainflammation**	new definition of subtype of low-grade inflammation based on experimental research; special case of CNS	intriguing proposal from basic research; might importantly contribute for new clinical grading of inflammation incl. neuroinflammation in mental disorders; experimental evidence	a priori difficult to prove as clinical entity
**Stress-induced Parainflammation**	parainflammation triggered by systemic stress	*see parainflammation*	*see parainflammation*
**Neuroprogression**	course characteristics in subgroups of severe mental illness associated with neuroinflammatory markers	intriguing proposal supported by clinical and experimental evidence	a priori difficult to prove as clinical entity
**Neurodegeneration/ Neuroinflammation**	any type of degenerative resp. inflammatory aspects/findings in central or peripheral nervous system	well-established concepts in basic research and clinic; differentiation between inflammation vs. degeneration	in clinical approach to diseased individuals sometimes difficult to realize/assess because of overlaps
**Special Case: Multiple Sclerosis (MS)**	autoimmune CNS disease, characterized by acute neuroinflammatory phases and neurodegeneration over the longer course	well-investigated disease; differential diagnostics of similar entities emerging	aspects of neurodegeneration long attributed to acute phase pathology, only recently being partially understood

Box 1 **This proposal of mild encephalitis (ME) subtypes is thought to provide a theoretical framework for a refined clinical differential diagnosis of relatively milder forms of neuroinflammation as compared to “classical” encephalitis, proposed to term ME. The proposed etiology-focused refined differential diagnostic framework of various types of ME is expected to become clinically relevant for improved causality-focused individualized treatment approaches and for further research. The references indicated represent a select choice only; more references to be found in the text.**

**Table:** 

**Differential Diagnostic Schedule of Mild Encephalitis (ME)/Mild Neuroinflammatory Subtypes – A Proposal from a clinical perspective** **Autoimmune mild neuroinflammation** -Autoimmune Psychosis (AP) with presence of CNS antibodies according to recent international consensus criteria (20). A distinction is made between possible AP (clinical syndrome which should lead to a broad organic diagnostic work-up), probable AP (with additional diagnostic findings such as pleocytosis in cerebrospinal fluid), and definite AP (with detection of antineuronal IgG antibodies in cerebrospinal fluid). -Poorly defined psychiatric cases of autoimmune origin without detection of (known) CNS antibodies, based on different diagnostic findings including brain biopsy pathologies or plausibility from therapy response on immune modulatory treatments (24, 28) **Infectious mild neuroinflammation** -Low-grade “encephalitis” caused by various neurotropic viruses or other infectious agents presenting with acute though mild neuroinflammation without neurological hard signs but prominent or exclusive psychiatric syndromes in context with paraclinical, especially intrathecal signs of specific infections (using PCR, antibody index, and culture). Historical examples are late-stage syphilis or influenza-associated severe mental disorders; recent examples include acute Lyme Neuroborreliosis, Bartonellosis, mild cerebral Whipple's disease, or potentially acute BDV infection [compare, for example, (39)] **Combined infectious-autoimmune mild neuroinflammation** -Persistent infection having induced in parallel an autoimmune response, pathology from both mechanisms can be involved in the pathogenesis of clinical syndrome observed in the diseased individual, paradigmatic cases represented by pediatric autoimmune neuropsychiatric disorder associated with streptococcal infection (PANDAS)/pediatric acute-onset neuropsychiatric syndrome (PANS), implicating requirement of primary agent-focused (elimination) and/or immune modulatory treatment (67). Besides PANDAS/PANS, the concept is also well established for anti-NMDAR encephalitis after herpes encephalitis (53, 56). **Multiconditional mild neuroinflammation** -Various risk factors acting together in rather balanced weight of each single factor, e.g., ubiquitous persistent infections plus social stress in context with genetic factors, and other variant endogenous factors (e.g., endocrine and immune status), age, etc. (18).

## Overlap, Differences, and Intermediate States Between Neuroinflammation and Neurodegeneration

The authors of a recent insightful review preferred to clearly distinguish neuroinflammation and neurodegeneration: they stated that neuroinflammatory disorders featured cytokines are produced by tissue-invading leukocytes while neurodegenerative disorders featured cytokines are produced by CNS-resident cells (78) (see also above), but how such difference can be assessed in clinical approach remains open. Thus, in clinical reality even when assessed in the individual patient, it may usually remain unclear whether pathologically-altered cytokine networks have beneficial or detrimental effects (see also above).

However, intermediate states or mild neuroinflammatory states probably prevail and appear to be clinically relevant for SMDs. Neuroinflammation can lead to neurodegeneration, milder forms of neuroinflammation can be observed in primary or seemingly primary neurodegenerative diseases. Such long discussed and in principle accepted example is neuroinflammation in Alzheimer`s disease (104–107), for which some discuss an infectious cause (108). Multiomic approaches are probed to identify the change of neuroinflammatory pathology over time in Alzheimer`s disease (106). Another example is Parkinson’s disease, to featuring complicated interactions and developments of variant oxidative stress and neuroinflammation during disease progression, under others attempted to unravel by integrative analysis of blood metabolomics and PET imaging in parallel (109). Multiple sclerosis, the best-investigated autoimmune neurological disease, features acute inflammation; however, this does only partly explain the observed brain atrophy, leading to a paradigm change and the search for and beginning implementation of new therapies focusing on neurodegeneration [compare for example (110)].

These examples just showcase recent approaches and limitations of insight on intermediate states and relations of neuroinflammation and neurodegeneration and their possible clinical relevance. Valid assessment and consented categorization of such intermediate states *in vivo* is just emerging. From a clinical perspective, it appears important to identify especially such low-grade or mild states of neuroinflammation in SMDs, because of possible important therapeutic implication, given a considerable subgroup of SMDs being therapy resistant to established treatments. For a practical perspective herein some details in recent diagnosis and therapy of AP are outlined.

## Clinical Diagnosis of Neuroinflammation in AP

Clinical detection of neuroinflammatory processes generally requires a multimodal approach. Diagnoses of AE or AP strongly recommend to include CSF examination (21, 51). Generally speaking, clinical assessment of inflammation represents a domain of blood examinations, whereas assessment of neuroinflammation requires usually CSF examination in combination with neuroimaging. This important differentiation was in research on SMDs often under-recognized.

### Blood

An appropriate method for the diagnosis of neuroinflammation *in vivo* using blood examination (e.g., measuring the C-reactive protein) is not available, including for cases of severe classical encephalitis, which require neuroimaging and CSF examination (44, 111, 112). Nevertheless, the detection of high titers of antineuronal antibodies in serum may be an indication of AE/AP (21), which is however not sufficient for diagnosis.

### Cerebrospinal Fluid (CSF)

The CSF examination remains a very important and, for many cases, the most sensitive method for the diagnosis of any type of neuroinflammation (44, 45, 112). CSF testing allows for the detection of acute neuroinflammation with increased white blood cell (WBC) counts (reference < 5/µl). Additionally, the number of WBCs reveals information about possible pathogens. Protein concentration (reference <450 mg/L) and age-dependent albumin quotients ([4 + age/15] × 10^–3^) are biomarkers for the blood–CSF barrier (BCSFB) (113–115). CSF-specific oligoclonal bands (OCBs) are markers for intrathecal immunoglobulin (Ig) G synthesis. Increased antibody indices (AIs) for pathogens or antineuronal antibodies reveal intrathecal antibody synthesis [including for anti-NMD-R antibodies (116)]. A positive MRZ reaction, however, reveals a polyclonal immune activation that can be often found in patients with multiple sclerosis (117, 118). With regard to testing of antineuronal antibodies in serum and CSF, heterogeneity exists between different methods (119). In affective and psychotic patient cohorts, a small subgroup of SMD cases demonstrate established signs of neuroinflammation in CSF, another large subgroup of overall up to 50-70% some minor CSF aberrancies (120–122). This phenomenon is currently difficult to explain, but it could be associated with some type of mild neuroinflammation [compare (120)]. Consensus criteria of AP diagnosis include CSF examination (20).

Apparently, there remain many challenges of improved CSF examination in SMDs: interpreting and handling cases with neuroinflammatory CSFs (e.g., with CSF specific OCBs) not matching AE or AP consensus criteria; background of possible relevant minor CSF aberrancies in SMDs [e.g., signs of blood-CSF barrier disturbance; neopterin increase, cytokine increases/decreases, signs of activated CSF lymphocytes or macrophages (120, 123–125)]; improving the sensitivity of CSF analysis methods and implementing these methods for routine use in psychiatric research to achieve improved diagnosis of mild forms of neuroinflammation with relevance for SMDs; detecting novel antineuronal antibodies (e.g., by tissue-based assays with indirect immunofluorescence on fixed/unfixed murine brain tissue) in patients at risk of AE/AP (126).

### Electroencephalography (EEG)

EEG is very sensitive for the detection of inflammatory brain processes (116). In AE patients, a specific EEG phenomenon—the so-called extreme delta brush—was observed (127). Further research is required to analyze whether comparable phenomena can also occur in other clearly defined AE/AP syndromes. The main challenge in EEG diagnostics remains in the principle of non-specificity. Multiple causes may result in a uniform end product. Therefore, EEG is currently used to support a multimodal clinical approach to AE/AP (116, 128).

### Neuroimaging

Various methods of neuroimaging are used, often even several methods simultaneously, for the diagnosis of AE/AP/ME. Here, we review some of these methods in brief from the viewpoint of rapidly evolving methods. MRI is currently the most important structural imaging method for the diagnosis of any type of suspected neuroinflammation, but it is not highly sensitive except for specific disorders like multiple sclerosis. Routine T2 or fluid-attenuated inversion recovery (FLAIR)-weighted images can show pathological processes. White matter lesions can be detected in the context of different autoimmune, infectious, metabolic, or other psychiatric disorders (e.g., multiple sclerosis shows typical periventricular or juxta-cortical lesions) (129). In limbic encephalitis, increased signal intensity of the mesiotemporal structures can be detected (51). In cases of blood–brain barrier dysfunction, T1-weighted images are sensitive to contrast-enhancing pathological processes. Diffusion-weighted imaging (DWI) is used to identify (sub)-acute infarcts. MR angiography can be used for vascular imaging (129).

However, novel methods could assist in gaining further insights into inflammatory brain processes. Resting-state fMRI can help detect disturbances in the functional connectivity of brain networks caused by inflammatory processes. In the case of anti-NMDA-R encephalitis, researchers have described characteristic alterations of whole-brain functional connectivity (130); this is of particular interest because structural MRI is inconspicuous in approximately two-thirds of anti-NMDA-R encephalitis cases (131). MR spectroscopy can detect different neurometabolites (e.g., choline), and most importantly, it allows for the noninvasive detection of glutamate and GABA levels in different brain regions, which might be especially interesting in cases of SMD patients with antibodies against glutamate or GABA receptors (132). The use of dynamic contrast enhanced (DCE) MRI, using t1 sequences, might support to better detect subtle blood–brain barrier dysfunction. The use of this method could help us see, for example, that disturbed BBB permeability can indicate a transition from optic neuritis to multiple sclerosis (133) and beyond in other pathologies. Diffusion tensor imaging (DTI) allows to describe fiber connections among different brain areas (134). Studies on patients with anti-NMDAR encephalitis have detected widespread white matter alterations (135). In everyday clinical practice, the combined use of various MRI methods (“multimodal imaging”) may lead to improved detection of different inflammatory profiles. Cerebral (^18^F) fluorodeoxyglucose positron emission tomography (FDG PET) imaging can detect inflammatory and neurodegenerative metabolic patterns in the CNS (136). Regarding the detection of AE, FDG–PET might be more sensitive than structural MRI (137). A whole-body FDG PET can be used for tumor screening in patients who present with paraneoplastic antibodies (128). Translocator protein 18 kDa (TSPO) PET imaging allows insights into microglial activation (136).

Challenges in the **neuroimaging** of AE/AP/ME are considerable despite recent progress from the use of multimodal approaches described above, as the sensitivity of established single neuroimaging methods is surprisingly limited, even in classical encephalitis, but new methods are being developed or probed, the results and outcome are awaited with great interest, especially multimodal neuroimaging seems to provide both more global and regional insight into pathological neuroinflammatory processes involving the brain.

### Brain Biopsy

The use of brain biopsy for potential AE/AP/ME cases is has rarely been reported in the literature, but biopsy provides an exceptional opportunity to sensitively prove mild neuroinflammation, provided the biopsy region is well-chosen (26). In addition, the specific type of neuroinflammation and detailed (micro-) localization in the tissue can be analyzed. However, the use of brain biopsy for diagnosis of AE/AP/ME is rather limited for ethical reasons from a risk–benefit viewpoint (24).

### Summary

Currently, for detecting AE/AP, a combined multimodal diagnostic approach with blood and CSF tests (including testing for antineuronal antibodies), EEG, MRI, and possibly FDG-PET is recommended (20, 51). In the case of clinical red flags and suggestive diagnostic findings (21, 138), a multidisciplinary approach would be desirable for individual patients with possible autoimmune-mediated SMDs. The situation is much less clear in the case of isolated abnormal CSF findings (e.g., in patients with isolated CSF specific OCBs or increased albumin quotients). Obviously, further research is needed here.

## Limitations

This was not an exhaustive review of the complex scenario of psycho-neuro-immunology in SMDs.

The author did also not intend to provide a clear roadmap for research on the ME hypothesis (if such was possible), but to highlight the scenario and clinical context associated with a number of *a priori* difficulties, which may be underscored in a prevailing research philosophy of preferring rapid clear “mechanistic” insights (139). Gaining further insight into these complex issues will be difficult, requiring time-consuming research and interdisciplinary approaches, especially together with clinical neurology, radiology, immunology and basic scientists.

One recent teaching example of surprising rapid progress by such approaches despite the many difficulties was the big California encephalitis project in children and adolescents, identifying various infectious etiologies of lymphocytic meningoencephalitis involving about 50% of cases; the mystery was solved 6 years later, when stored material was reanalyzed for anti-NMDAR antibodies and AE was confirmed in nearly all of the left open cases (140). The recent example of human BDV infection coming just in focus again was outlined in some detail (see above), the difficulties and limitations of available clinical methods considering the possibility of milder (non-deadly) forms of BoDV-1 disease becoming evident.

## Conclusion

There is now clear evidence that a small subgroup of patients with SMDs can be diagnosed as AE/AP (128). For predominant psychotic cases, not necessarily fulfilling the criteria for AE, consensus criteria for AP were very recently established (21). AP cases would also fulfil the criteria of ME, as defined earlier. Another subgroup of similar cases that do not present CNS autoantibodies (and thus cases do not fulfill the proposed criteria of definite AP instead of possible AP according to Najjar et al. (24) appears to emerge, also matching the ME hypothesis, diagnosed with brain biopsy or multimodal and new (eg. tissue –based antibody testing) approaches in specialized centers including complicated differential diagnosis (28–31, 126, 141, 142); some of these cases may similarly respond to aggressive immune treatments like AE and AP cases, but may remain suggestive or “possible” cases. Plausibly, such cases without presenting CNS antibodies, at least with regard to presently known antibodies, may be related to undefined immune pathology, e.g. CSF cell activation is not routinely specified only in rare research studies [compare (125)], or speculatively from other neuroinflammatory mechanisms, maybe including such triggered from brain vasculopathy due to genetic liability [compare (143)] or immune developmental factors including early and later infections (144–148) and other pathomechanisms in complex neuro-psycho-immunological scenario. Not all questions can be cleared by experimental research, instead there is also a justification and even need of careful designed experimental clinical approaches including experimental therapies [compare also (71, 149)]. Multimodal group studies including neuroimaging can provide a general basis especially when combined with immune-inflammatory markers (150–153). Unfortunately CSF studies are still rare in research on SMDs but their extraordinary relevance increasingly recognized (71, 154), because can still provide the best and most specific clinical information about neuroinflammatory processes.

However, carefully considered intervention trials should be continued in specialized centers only, whereas general hospitals should rely on conservative approach along consensus criteria (21, 91). In individual patients, off-label treatment approaches can be very successful and helpful in understanding details of neuroinflammatory constellations by indirect reasoning (compare the example of tertiary syphilis), when followed and analyzed under strict rules of clinical research. Difficulties persist in differentiating innocent cases of CNS autoantibody prevalence (when tested in blood) from relevant ones (102), which is however similarly true for antibodies against infectious agents. Thus, only multimodal clinical approaches combined with basic and experimental research will together be able to develop criteria for the differentiation of clinically relevant cases of mild neuroinflammation in SMDs, a recognized challenge in the emerging precision medicine of SMDs (63). Psychiatrists should learn improved “organic” neuro-psycho-immuno diagnostics in interaction with other disciplines and from emerging psycho-neuro-immunology research, similarly claimed by a panel of other experts (71).

## Author Contributions

The author confirms being the sole contributor of this work and has approved it for publication.

## Conflict of Interest

The author declares that the research was conducted in the absence of any commercial or financial relationships that could be construed as a potential conflict of interest.

## References

[1] Anderson G Berk M Dodd S Bechter K Altamura AC Kanba S Immuno-inflammatory, oxidative and nitrosative stress, and neuroprogressive pathways in the etiology, course and treatment of schizophrenia. Prog Neuropsychopharmacol Biol Psychiatry (2013) 42:1–4. 10.1016/j.pnpbp.2012.10.008 23085074

[2] Halaris A Neuroinflammation and neurotoxicity contribute to neuroprogression in neurological and psychiatric disorders. Future Neurol (2018) 13(2):59–69. 10.2217/fnl-2017-0039

[3] Halaris A Bechter K Haroon E Leonard BE Miller A Pariante C The Future of Psychoneuroimmunology: Promises and Challenges. In: Javed A Fountoulakis KN, editors. Advances in Psychiatry. Cham: Springer International Publishing (2019). p. 235–66. 10.1007/978-3-319-70554-5_15

[4] Najjar S Pearlman DM Neuroinflammation and white matter pathology in schizophrenia: Systematic review. Schizophr Res (2015) 161(1):102–12. 10.1016/j.schres.2014.04.041 24948485

[5] Najjar S Pearlman DM Alper K Najjar A Devinsky O Neuroinflammation and psychiatric illness. J Neuroinflamm (2013) 10:43. 10.1186/1742-2094-10-43 PMC3626880 23547920

[6] Liew FY Girard J-P Turnquist HR Interleukin-33 in health and disease. Nat Rev Immunol (2016) 16(11):676–89. 10.1038/nri.2016.95 27640624

[7] Cadwell K Crosstalk between autophagy and inflammatory signalling pathways: balancing defence and homeostasis. Nat Rev Immunol (2016) 16(11):661–75. 10.1038/nri.2016.100 PMC5343289 27694913

[8] Zöller M Janus-Faced Myeloid-Derived Suppressor Cell Exosomes for the Good and the Bad in Cancer and Autoimmune Disease. Front Immunol (2018) 9:137:137. 10.3389/fimmu.2018.00137 29456536 PMC5801414

[9] Drexhage HA Editorial: Activation and de-activation of inflammatory pathways. The disequilibrium of immune-neuro-endocrine networks in psychiatric disorders. Brain Behav Immun (2019) 78:5–6. 10.1016/j.bbi.2019.01.007 30682504

[10] Bechter K Bipolar cells precede bipolar minds - But relations are complex. Brain Behav Immun (2016) 58:9–10. 10.1016/j.bbi.2016.07.005 27401685

[11] Benedetti F Aggio V Pratesi ML Greco G Furlan R Neuroinflammation in Bipolar Depression. Front Psychiatry (2020) 11:71. 10.3389/fpsyt.2020.00071 32174850 PMC7054443

[12] Cuthbert BN Insel TR Toward the future of psychiatric diagnosis: the seven pillars of RDoC. BMC Med (2013) 11:126. 10.1186/1741-7015-11-126 23672542 PMC3653747

[13] Steiner J Bogerts B Sarnyai Z Walter M Gos T Bernstein H-G Bridging the gap between the immune and glutamate hypotheses of schizophrenia and major depression: Potential role of glial NMDA receptor modulators and impaired blood-brain barrier integrity. World J Biol Psychiatry (2012) 13(7):482–92. 10.3109/15622975.2011.583941 21707463

[14] Bernstein H-G Steiner J Guest PC Dobrowolny H Bogerts B Glial cells as key players in schizophrenia pathology: Recent insights and concepts of therapy. Schizophr Res (2015) 161(1):4–18. 10.1016/j.schres.2014.03.035 24948484

[15] Cai HQ Catts VS Webster MJ Galettli C Liu D O`Donell M Increased macrophages and changed brain endothelial cell gene expression in the frontal cortex of people with schizophrenia displaying inflammation. Mol Psychiatry (2018) 25, 761–75. 10.1038/s41380-018-0235-x PMC7156343 30214039

[16] Bogerts B Winopal D Schwarz S Schlaaff K Dobrowolny H Mawrin C Evidence of neuroinflammation in subgroups of schizophrenia and mood disorder patients: A semiquantitative postmortem study of CD3 and CD20 immunoreactive lymphocytes in several brain regions. Neurol Psychiatry Brain Res (2017) 23:2–9. 10.1016/j.npbr.2016.11.001

[17] Bechter K Mild encephalitis underlying psychiatric disorders-a reconsideration and hypothesis exemplified on Borna disease. Neurol Psychiatry Brain Res (2001) (9):55–70.

[18] Bechter K Updating the mild encephalitis hypothesis of schizophrenia. Prog Neuropsychopharmacol Biol Psychiatry (2013) 42:71–91. 10.1016/j.pnpbp.2012.06.019 22765923

[19] Graus F Dalmau J CNS autoimmunity: New findings and pending issues. Lancet Neurol (2012) 11(1):17–9. 10.1016/S1474-4422(11)70280-0 PMC3723139 22172617

[20] Pollak TA Pruess H Tebartz van Elst L Vincent A Najjar S Bechter K Autoimmune Psychosis. Correspondence: Authors reply. Lancet Psychiatry (2020) (7):122–5. 10.1016/S2215-0366(19)30527-9 31981531

[21] Pollak TA Lennox BR Müller S Benros ME Pruess H Tebartz van Elst L Autoimmune psychosis: an international consensus on an approach to the diagnosis and management of psychosis of suspected autoimmune origin. Lancet Psychiatry (2020) 7:93–108. 10.1016/S2215-0366(19)30290-1 31669058

[22] Al-Diwani A Pollak TA Langford AE Lennox BR Synaptic and Neuronal Autoantibody-Associated Psychiatric Syndromes: Controversies and Hypotheses. Front Psychiatry (2017) 8:1–11. 10.3389/fpsyt.2017.00013 28220082 PMC5292436

[23] Bechter K Deisenhammer F Psychiatric syndromes other than dementia. Handb Clin Neurol (2017) 146:285–96. 10.1016/B978-0-12-804279-3.00017-4 29110776

[24] Najjar S Steiner J Najjar A Bechter K A clinical approach to new-onset psychosis associated with immune dysregulation: the concept of autoimmune psychosis. J Neuroinflamm (2018) 15(1):40. 10.1186/s12974-018-1067-y PMC5809809 29433523

[25] Najjar S Pahlajani S Sanctis V de Stern JNH Najjar A Chong D Neurovascular Unit Dysfunction and Blood–Brain Barrier Hyperpermeability Contribute to Schizophrenia Neurobiology: A Theoretical Integration of Clinical and Experimental Evidence. Front Psychiatry (2017) 8:1–11. 10.3389/fpsyt.2017.00083 28588507 PMC5440518

[26] Najjar S Pearlman DM Hirsch S Friedman K Strange J Reidy J Brain biopsy findings link major depressive disorder to neuroinflammation, oxidative stress, and neurovascular dysfunction: A case report. Biol Psychiatry (2014) 75(12):e23–6. 10.1016/j.biopsych.2013.07.041 24075735

[27] Najjar S Pearlman D Zagzag D Golfinos J Devinsky O Glutamic acid decarboxylase autoantibody syndrome presenting as schizophrenia. Neurologist (2012) 18(2):88–91. 10.1097/NRL.0b013e318247b87d 22367838

[28] Endres D Perlov E Feige B Altenmüller D-M Venhoff N van Tebartz Elst L Schizophrenia Associated with Epileptiform Discharges without Seizures Successfully Treated with Levetiracetam. Front Psychiatry (2017) 8:1–5. 10.3389/fpsyt.2017.00012 28228737 PMC5296318

[29] Endres D Perlov E Stich O Meyer PT Lützen N van Tebartz Elst L Case report: Low-titre anti-Yo reactivity in a female patient with psychotic syndrome and frontoparieto-cerebellar atrophy. BMC Psychiatry (2015) 15:1–5. 10.1186/s12888-015-0486-x 25963777 PMC4436095

[30] Endres D Perlov E Riering AN Maier V Stich O Dersch R Steroid-Responsive Chronic Schizophreniform Syndrome in the Context of Mildly Increased Antithyroid Peroxidase Antibodies. Front Psychiatry (2017) 8:1–6. 10.3389/fpsyt.2017.00064 28484400 PMC5399039

[31] Endres D Vry MS Dykierek P Riering AN Lüngen E Stich O Plasmapheresis Responsive Rapid Onset Dementia with Predominantly Frontal Dysfunction in the Context of Hashimoto’s Encephalopathy. Front Psychiatry (2017) 8:1–7. 10.3389/fpsyt.2017.00212 29123489 PMC5662557

[32] Bechter K Borna Disease Virus: Mögliche Ursache neurologischer und psychiatrischer Störungen des Menschen. Steinkopff: Darmstadt (1998).

[33] Bechter K Herzog S Fleischer B Schüttler R Rott R Magnetic resonance imaging in psychiatric patients with and without serum antibodies against Borna disease. Nervenarzt (1987) (58):617–24. 3120021

[34] Bechter K Herzog S Behr W Schüttler R Investigations of cerebrospinal fluid in Borna disease virus seropositive psychiatric patients. Eur Psychiatry (1995) 10(5):250–8. 10.1016/0924-9338(96)80302-6 19698348

[35] Bechter K Herzog S Schuettler R Borna disease virus- possible causal agent in psychiatric and neurological disorders in two families. Psychiatry Res (1992) (42):291–4. 10.1016/0165-1781(92)90121-I 1496060

[36] Richt JA Pfeuffer I Christ M Frese K Bechter K Herzog S Borna disease virus infection in animals and humans. Em Infect Dis (1997) 3(3):343–52. 10.3201/eid0303.970311 PMC2627631 9284379

[37] Herzog S Molecular characterization of Borna disease virus from naturally infected animals and possible links to human disorders. Arch Virol (1997) 13(Suppl):183–90. 10.1007/978-3-7091-6534-8_17 9413537

[38] Rott R Herzog S Fleischer B Winokur A Amsterdam J Dyson W Detection of serum-antibodies to Borna disease virus in patients with psychiatric disorders. Science (1985) 228:755–6. 10.1126/science.3922055 3922055

[39] Rott R Herzog S Bechter K Frese K Borna disease, a possible hazard for man? Arch Virol (1991) 118:143–9. 10.1007/BF01314025 2069502

[40] Rott O Herzog S Cash E T cell memory specific for self and non-self antigens in rats persistently infected with Borna disease virus. Clin Exp Immunol (1993) 93:370–6. 10.1111/j.1365-2249.1993.tb08187.x PMC1554921 7690314

[41] Korn K Coras R Bobinger T Herzog SM Luecking H Stoehr R Fatal Encephalitis Associated with Borna Disease Virus 1. N Engl J Med (2018) 379(14):1375–7. 10.1056/NEJMc1800724 30281979

[42] Schlottau K Forth L Angstwurm K Hoeper D Zecher D Liesche F Fatal Encephalitic Borna Disease Virus 1 in Solid-Organ Transplant Recipients. N Engl J Med (2018) 379(14):1377–9. 10.1056/NEJMc1803115 30281984

[43] Niller HH Angstwurm K Rubbenstroth D Schlottau K Ebinger A Giese S Zoonotic spillover infections with Borna disease virus 1 leading to fatal human encephalitis, 1999–2019: an epidemiological investigation. Lancet Infect Dis (2020) 20(4):467–77. 10.1016/S1473-3099(19)30546-8 31924550

[44] Venkatesan A Tunkel AR Bloch KC Lauring AS Sejvar J Bitnun A Case definitions, diagnostic algorithms, and priorities in encephalitis: Consensus statement of the international encephalitis consortium. Clin Infect Dis (2013) 57(8):1114–28. 10.1093/cid/cit458 PMC3783060 23861361

[45] Deisenhammer F Teunissen CE Tumani H Cerebrospinal fluid in neurologic disorders. Elsevier: Amsterdam, Kidlington, Cambridge, MA (2017).

[46] Wollinsky KH CSF filtration is an effective treatment of Guillain-Barre syndrome: a randomized clinical trial. Neurology (2001) (57):774–80. 10.1212/WNL.57.5.774 11552002

[47] Bechter K Borna disease virus-related therapy-resistant depression improved after cerebrospinal fluid filtration. J Psychiatr Res (2000) (34):393–6. 10.1016/S0022-3956(00)00033-9 11165306

[48] Bechter K Herzog S Schreiner V Wollinsky KH Schüttler R Cerebrospinal fluid filtration in a case of schizophrenia related to ‘subclinical’ Borna disease virus encephalitis. In: Müller N, editor. Psychiatry, Psychoneuroimmunology and Viruses. Key Top Brain Res Wien. (1999). p. 19–35 19–35.

[49] Bechter K Schreiner V Herzog S Breitinger N Wollinsky KH Brinkmeier H Liquorfiltration als experimentelle Therapie bei therapieresistenten Psychosen Borna-Disease-Virus-seropositiver Patienten. Psychiatr Prax (2003) 30(Suppl 2):216–20. 10.1055/s-2003-39747 13130379

[50] Bechter K Herzog S Schreiner V Wollinsky KH Schüttler R Cerebrospinal fluid filtration in Borna-disease-virus-encephalits-related schizophrenia: A new therapeutic perspective? Neurol Psychiatry Brain Res (1998) (6):85–6.

[51] Graus F Titulaer MJ Balu R Benseler S Bien CG Celucci T A clinical approach to diagnosis of autoimmune encephalitis. Lancet Neurol (2016) 15(4):391–404. 10.1016/S1474-4422(15)00401-9 26906964 PMC5066574

[52] Kreye J Wenke NK Chayka M Leubner J Murugan R Maier M Human cerebrospinal fluid monoclonal N-methyl-D-aspartate receptor autoantibodies are sufficient for encephalitis pathogenesis. Brain (2016) 139(Pt 10):2641–52. 10.1093/brain/aww208 27543972

[53] Dalmau J Autoimmunity: The good, the bad, and the ugly. Neurol Neuroimmunol Neuroinflamm (2015) 2(6):1–3. 10.1212/NXI.0000000000000181 PMC4674432 26668820

[54] Coutinho E Harrison P Vincent A Do neuronal autoantibodies cause psychosis? A neuroimmunological perspective. Biol Psychiatry (2014) 75(4):269–75. 10.1016/j.biopsych.2013.07.040 24090795

[55] Dalmau J Tüzün E Wu H Masjuan J Rossi JE Voloschin A Paraneoplastic Anti–N-methyl-D-aspartate Receptor Encephalitis Associated with Ovarian Teratoma. Ann Neurol (2007) 61(1):25–36. 10.1002/ana.21050 17262855 PMC2430743

[56] Prüss H Postviral autoimmune encephalitis: manifestations in children and adults. Curr Opin Neurol (2017) 30(3):327–33. 10.1097/WCO.0000000000000445 28234798

[57] Dalmau J NMDA receptor encephalitis and other antibody-mediated disorders of the synapse. Neurology (2016) 87:2471–82. 10.1212/WNL.0000000000003414 PMC5177671 27920282

[58] Zuliani L Zoccarato M Open issues in antibody-mediated encephalitis. Future Neurol (2020) 15(2):FNL40. 10.2217/fnl-2019-0026

[59] Riedmüller R Müller S Ethical Implications of the Mild Encephalitis Hypothesis of Schizophrenia. Front Psychiatry (2017) 8:1–14. 10.3389/fpsyt.2017.00038 28348532 PMC5346578

[60] Bechter K Research strategies in ‘slow’ infections in psychiatry_History of Psychiatry. History Psychiatry (1995) (6):503–11. 10.1177/0957154X9500602407 11609007

[61] Müller N Bechter K The mild encephalitis concept for psychiatric disorders revisited in the light of current psychoneuroimmunological findings. Neurol Psychiatry Brain Res (2013) 19(3):87–101. 10.1016/j.npbr.2013.04.004

[62] Zusso M Stokes L Moro S Giusti P Editorial: Neuroinflammation and Its Resolution: From Molecular Mechanisms to Therapeutic Perspectives. Front Pharmacol (2020) 11:1–4. 10.3389/fphar.2020.00480 32322215 PMC7156608

[63] Fernandes BS Borgwardt S Carvalho AF Steiner J Editorial: Back to the Future: On the Road Towards Precision Psychiatry. Front Psychiatry (2020) 11:112. 10.3389/fpsyt.2020.00112 32174859 PMC7054451

[64] Halaris A Leonard BE Unraveling the complex interplay of immunometabolic systems that contribute to the neuroprogression of psychiatric disorders. Neurol Psychiatry Brain Res (2019) 32:111–21. 10.1016/j.npbr.2019.05.005

[65] Berk M Kapczinski F Andreazza AC Dean OM Giolandeo F Maes M Pathways underlying neuroprogression in bipolar disorder: focus on inflammation, oxidative stress and neurotrophic factors. Neurosci Biobehav Rev (2011) 35(3):804–17. 10.1016/j.neubiorev.2010.10.001 20934453

[66] Swedo SE Seidlitz J Kovacevic M Latimer ME Hommer R Lougee L Clinical presentation of pediatric autoimmune neuropsychiatric disorders associated with streptococcal infections in research and community settings. J Child Adolesc Psychopharmacol (2015) 25(1):26–30. 10.1089/cap.2014.0073 25695941 PMC4340334

[67] Swedo SE Frankovich J Murphy TK Overview of Treatment of Pediatric Acute-Onset Neuropsychiatric Syndrome. J Child Adolesc Psychopharmacol (2017) 27(7):562–5. 10.1089/cap.2017.0042 PMC5610386 28722464

[68] Bechter K Bindl A Horn M Schreiner V Therapieresistente Depression mit Fatigue. Fall einer vermutlichen streptokokkenassoziierten Autoimmunkrankheit. Der Nervenarzt (2007) 78(3):338–41. 10.1007/s00115-006-2178-8 17160540

[69] Platt MP Agalliu D Cutforth T Hello from the Other Side: How Autoantibodies Circumvent the Blood–Brain Barrier in Autoimmune Encephalitis. Front Immunol (2017) 8:1–15. 10.3389/fimmu.2017.00442 28484451 PMC5399040

[70] Breitschwerdt EB Greenberg R Maggi RG Mozayeni BR Lewis A Bradley JM Bartonella henselae Bloodstream Infection in a Boy With Pediatric Acute-Onset Neuropsychiatric Syndrome. J Cent Nerv Syst Dis (2019) 11:1179573519832014. 10.1177/1179573519832014 30911227 PMC6423671

[71] Caldwell LJ Subramaniam S MacKenzie G Shah DK Maximising the potential of neuroimmunology. Brain Behav Immun (2020) 87:189–92. 10.1016/j.bbi.2020.03.010 PMC8353661 32201255

[72] Medzhitov R Origin and physiological roles of inflammation. Nature (2008) 454(7203):428–35. 10.1038/nature07201 18650913

[73] Wohleb ES McKim DB Sheridan JF Godbout JP Monocyte trafficking to the brain with stress and inflammation: A novel axis of immune-to-brain communication that influences mood and behavior. Front Neurosci (2014) 8:447. 10.3389/fnins.2014.00447 25653581 PMC4300916

[74] Berk M Neuroprogression: pathways to progressive brain changes in bipolar disorder. Int J Neuropsychopharmacol (2009) 12(4):441–5. 10.1017/S1461145708009498 18922203

[75] Davis J Moylan S Harvey BH Maes M Berk M Neuroprogression in schizophrenia: Pathways underpinning clinical staging and therapeutic corollaries. Aust N Z J Psychiatry (2014) 48(6):512–29. 10.1177/0004867414533012 24803587

[76] Davis J Maes M Andreazza A McGrath JJ Tye SJ Berk M Towards a classification of biomarkers of neuropsychiatric disease: From encompass to compass. Mol Psychiatry (2015) 20(2):152–3. 10.1038/mp.2014.139 25349167

[77] Müller N Neuroprogression in Schizophrenia and Psychotic Disorders: The Possible Role of Inflammation. Mod Trends Pharmacopsychiatry (2017) 31:1–9. 10.1159/000470802 28738373

[78] Becher B Spath S Goverman J Cytokine networks in neuroinflammation. Nat Rev Immunol (2017) 17(1):49–59. 10.1038/nri.2016.123 27916979

[79] Frühbeis C Fröhlich D Krämer-Albers E-M Emerging roles of exosomes in neuron-glia communication. Front Physiol (2012) 3:119. 10.3389/fphys.2012.00119 22557979 PMC3339323

[80] Kawikova I Askenase PW Diagnostic and therapeutic potentials of exosomes in CNS diseases. Brain Res (2015) 1617:63–71. 10.1016/j.brainres.2014.09.070 25304360 PMC4862949

[81] Pusic AD Pusic KM Kraig RP What are exosomes and how can they be used in multiple sclerosis therapy? Expert Rev Neurother (2014) 14(4):353–5. 10.1586/14737175.2014.890893 PMC4090348 24552578

[82] Street JM Barran PE Mackay CL Weidt S Balmforth C Walsh T Identification and proteomic profiling of exosomes in human cerebrospinal fluid. J Transl Med (2012) 10:5. 10.1186/1479-5876-10-5 22221959 PMC3275480

[83] Bechter K The peripheral cerebrospinal fluid outflow pathway – physiology and pathophysiology of CSF recirculation: A review and hypothesis. Neurol Psychiatry Brain Res (2011) 17(3):51–66. 10.1016/j.npbr.2011.06.003

[84] Bechter K Hof PR Benveniste H On the flow dynamics of cerebrospinal fluid. Neurol Psychiatry Brain Res (2015) 21(2):96–103. 10.1016/j.npbr.2015.01.001 PMC4594867 26451075

[85] Benveniste H Hof PR Nedergaard M Bechter K Modern cerebrospinal fluid flow research and Heinrich Quincke’s seminal 1872 article on the distribution of cinnabar in freely moving animals. J Comp Neurol (2015) 523(12):1748–55. 10.1002/cne.23758 PMC4472498 25684428

[86] Haarman BCMB Burger H Doorduin J Renken RJ Sibelijn-Kuiper AJ Marsman JBC Volume, metabolites and neuroinflammation of the hippocampus in bipolar disorder - A combined magnetic resonance imaging and positron emission tomography study. Brain Behav Immun (2016) 56:21–33. 10.1016/j.bbi.2015.09.004 26348581

[87] Haarman BCMB Riemersma-Van der Lek RF de Groot JC Ruhe HGE Klein H Zandstra T Neuroinflammation in bipolar disorder - A (11)C-(R)-PK11195 positron emission tomography study. Brain Behav Immun (2014) 40:219–25. 10.1016/j.bbi.2014.03.016 24703991

[88] Gibney SM Drexhage HA Evidence for a dysregulated immune system in the etiology of psychiatric disorders. J Neuroimmune Pharmacol (2013) 8(4):900–20. 10.1007/s11481-013-9462-8 23645137

[89] Drexhage RC Hoogenboezem TH Versnel MA Berghout A Nolen WA Drexhage HA The activation of monocyte and T cell networks in patients with bipolar disorder. Brain Behav Immun (2011) 25(6):1206–13. 10.1016/j.bbi.2011.03.013 21443944

[90] Bechter K Encephalitis, Mild Encephalitis, Neuroprogression, or Encephalopathy-Not Merely a Question of Terminology. Front Psychiatry (2018) 9:782. 10.3389/fpsyt.2018.00782 30787887 PMC6372546

[91] Pollak TA Pruess H Tebartz van Elst L Vincent A Najjar S Bechter K Author’s reply. Lancet Psychiatry (2020) 7:123–5. 10.1016/S2215-0366(19)30527-9 31981531

[92] Zuliani L Graus F Giometto B Bien C Vincent A Central nervous system neuronal surface antibody associated syndromes: Review and guidelines for recognition. J Neurol Neurosurg Psychiatry (2012) 83(6):638–45. 10.1136/jnnp-2011-301237 PMC3348613 22448032

[93] Irani SR Bera K Waters P Zuliani L Maxwell S Zandi M N-methyl-d-aspartate antibody encephalitis: Temporal progression of clinical and paraclinical observations in a predominantly non-paraneoplastic disorder of both sexes. Brain (2010) 133(6):1655–67. 10.1093/brain/awq113 PMC2877907 20511282

[94] Dalmau J Lancaster E Martinez-Hernandez E Rosenfeld MR Balice-Gordon R Clinical experience and laboratory investigations in patients with anti-NMDAR encephalitis. Lancet Neurol (2011) 10(1):63–74. 10.1016/S1474-4422(10)70253-2 21163445 PMC3158385

[95] Armangue T Titulaer MJ Málaga I Bataller L Cabilondo I Graus F Pediatric Anti-NMDAR encephalitis-Clinical analysis and novel findings in a series of 20 patients. J Pediatr (2013) 162(4):850–6.e2. 10.1016/j.jpeds.2012.10.011 PMC3582718 23164315

[96] Armangue T Leypoldt F Dalmau J Auto-immune encephalitis as differential diagnosis of infectious encephalitis. Curr Opin Neurol (2014) 27(3):361–8. 10.1097/WCO.0000000000000087 PMC4132825 24792345

[97] Dalmau J The case for autoimmune neurology. Neurol Neuroimmunol Neuroinflamm (2017) 4(4):1. 10.1212/NXI.0000000000000373 PMC5499978 28702477

[98] Irani SR Gelfand JM Al-Diwani A Vincent A Cell-surface central nervous system autoantibodies: Clinical relevance and emerging paradigms. Ann Neurol (2014) 76(2):168–84. 10.1002/ana.24200 PMC4141019 24930434

[99] Lang K Prüss H Frequencies of neuronal autoantibodies in healthy controls: Estimation of disease specificity. Neurol Neuroimmunol Neuroinflamm (2017) 4(5):1–8. 10.1212/NXI.0000000000000386 PMC5515597 28761905

[100] Steiner J Schiltz K Bernstein H-G Bogerts B Antineuronal antibodies against neurotransmitter receptors and synaptic proteins in schizophrenia: Current knowledge and clinical implications. CNS Drugs (2015) 29(3):197–206. 10.1007/s40263-015-0233-3 25724386

[101] Hammer C Stepniak B Schneider A Papiol S Tantra M Begemann M Neuropsychiatric disease relevance of circulating anti-NMDA receptor autoantibodies depends on blood-brain barrier integrity. Mol Psychiatry (2014) 19(10):1143–9. 10.1038/mp.2013.110 23999527

[102] Pan H Steixner-Kumar AA Seelbach A Deutsch N Ronnenberg A Tapken D Multiple inducers and novel roles of autoantibodies against the obligatory NMDAR subunit NR1: a translational study from chronic life stress to brain injury. Mol Psychiatry (2020). 10.1038/s41380-020-0672-1 PMC8440197 32089545

[103] Hammer C Zerche M Schneider A Begemann M Nave K-A Ehrenreich H Apolipoprotein E4 carrier status plus circulating anti-NMDAR1 autoantibodies: Association with schizoaffective disorder. Mol Psychiatry (2014) 19(10):1054–6. 10.1038/mp.2014.52 PMC4195337 24888362

[104] Hensley K Neuroinflammation in Alzheimer’s Disease: Mechanisms, Pathologic Consequences, and Potential for Therapeutic Manipulation. J Alzheimers Dis (2010) 21(1):1–14. 10.3233/JAD-2010-1414 20182045 PMC3792565

[105] Marlatt MW Bauer J Aronica E van Haastert ES Hoozemans JJM Joels M Proliferation in the Alzheimer Hippocampus Is due to Microglia, Not Astroglia, and Occurs at Sites of Amyloid Deposition. Neural Plast (2014) 2014:1–12. 10.1155/2014/693851 PMC4157009 25215243

[106] Marttinen M Paananen J Neme A Mitra V Takalo M Natunen T A multiomic approach to characterize the temporal sequence in Alzheimer’s disease-related pathology. Neurobiol Dis (2019) 124:454–68. 10.1016/j.nbd.2018.12.009 30557660

[107] Shen Y Yang L Li R What does complement do in Alzheimer’s disease? Old molecules with new insights. Transl Neurodegener (2013) 2:21. 10.1186/2047-9158-2-21 24119446 PMC3853043

[108] Miklossy J Alzheimer’s disease - a neurospirochetosis. Analysis of the evidence following Koch’s and Hill’s criteria. J Neuroinflamm (2011) 8:90. 10.1186/1742-2094-8-90 PMC3171359 21816039

[109] Glaab E Trezzi J-P Greuel A Jäger C Hodak Z Drzezga A Integrative analysis of blood metabolomics and PET brain neuroimaging data for Parkinson’s disease. Neurobiol Dis (2019) 124:555–62. 10.1016/j.nbd.2019.01.003 30639291

[110] Chataway J de Angelis F Connick P Parker RA Plantone D Doshi A Efficacy of three neuroprotective drugs in secondary progressive multiple sclerosis (MS-SMART): a phase 2b, multiarm, double-blind, randomised placebo-controlled trial. Lancet Neurol (2020) 19:214–25. 10.1016/S1474-4422(19)30485-5 PMC7029307 31981516

[111] Wildemann B Reiber H Oschmann P Laboratory diagnosis in neurology. Stuttgart, editor. Thieme: New York (2010).

[112] Britton PN Eastwood K Paterson B Durrheim DN Dale RC Cheng AC Consensus guidelines for the investigation and management of encephalitis in adults and children in Australia and New Zealand. Intern Med J (2015) 45(5):563–76. 10.1111/imj.12749 25955462

[113] Reiber H Proteins in cerebrospinal fluid and blood Barriers, CSF flow rate and source-related dynamics. Restorative Neurol Neurosci (2003) 21(3–4):79–96. 14530572

[114] Reiber H Dynamics of brain-derived proteins in cerebrospinal fluid. Clin Chim Acta (2001) 310(2):173–86. 10.1016/S0009-8981(01)00573-3 11498083

[115] Stich O Rauer S Kaiser R Liquordiagnostik Neurologie compact. Für Klinik und Praxis: Liquordiagnostik. 6th Hufschmidt A Lücking CH Rauer S, editors. Stuttgart: Thieme Stuttgart (2013).

[116] van Tebartz Elst L Bechter K Prüss H Hasan A Steiner J Leypoldt F Autoantikörper-assoziierte schizophreniforme Psychosen: Pathophysiologie, Diagnostik und Therapie. Der Nervenarzt (2019) 90(7):745–61. 10.1007/s00115-019-0735-1 31197396

[117] Reiber H Polyspecific antibodies without persisting antigen in multiple sclerosis, neurolupus and Guillain-Barré syndrome: immune network connectivity in chronic diseases. Arq Neuropsiquiatr (2017) 75(8):580–8. 10.1590/0004-282X20170081 28813089

[118] Hottenrott T Dersch R Berger B Rauer S Huzly D Stich O The MRZ reaction in primary progressive multiple sclerosis. Fluids Barriers CNS (2017) 14:1–8. 10.1186/s12987-016-0049-7 28166789 PMC5294835

[119] Jézéquel J Rogemond V Pollak T Lepleux M Jacobson L Grea H Cell- and Single Molecule-Based Methods to Detect Anti-N-Methyl-D-Aspartate Receptor Autoantibodies in Patients With First-Episode Psychosis From the OPTiMiSE Project. Biol Psychiatry (2017) 82(10):766–72. 10.1016/j.biopsych.2017.06.015 28780967

[120] Bechter K CSF diagnostics in psychiatry – present status – future projects. Neurol Psychiatry Brain Res (2016) 22(2):69–74. 10.1016/j.npbr.2016.01.008

[121] Bechter K Reiber H Herzog S Fuchs D Tumani H Maxeiner HG Cerebrospinal fluid analysis in affective and schizophrenic spectrum disorders: Identification of subgroups with immune responses and blood-CSF barrier dysfunction. J Psychiatr Res (2010) 44(5):321–30. 10.1016/j.jpsychires.2009.08.008 19796773

[122] Endres D Perlov E Baumgartner A Hottenrott T Dersch R Stich O Immunological findings in psychotic syndromes: A tertiary care hospital’s CSF sample of 180 patients. Front Hum Neurosci (2015) 9:1–11. 10.3389/fnhum.2015.00476 26441585 PMC4564575

[123] Maxeiner H-G Marion Schneider E Kurfiss S-T Brettschneider J Tumani H Bechter K Cerebrospinal fluid and serum cytokine profiling to detect immune control of infectious and inflammatory neurological and psychiatric diseases. Cytokine (2014) 69(1):62–7. 10.1016/j.cyto.2014.05.008 25022963

[124] Kuehne LK Reiber H Bechter K Hagberg L Fuchs D Cerebrospinal fluid neopterin is brain-derived and not associated with blood-CSF barrier dysfunction in non-inflammatory affective and schizophrenic spectrum disorders. J Psychiatr Res (2013) 47(10):1417–22. 10.1016/j.jpsychires.2013.05.027 23790260

[125] Maxeiner H-G Rojewski MT Schmitt A Tumani H Bechter K Schmitt M Flow cytometric analysis of T cell subsets in paired samples of cerebrospinal fluid and peripheral blood from patients with neurological and psychiatric disorders. Brain Behav Immun (2009) 23(1):134–42. 10.1016/j.bbi.2008.08.003 18771722

[126] Endres D Prüss H Rauer S Süß P Venhoff N Feige B Probable Autoimmune Catatonia With Antibodies Against Cilia on Hippocampal Granule Cells and Highly Suspicious Cerebral FDG-Positron Emission Tomography Findings. Biol Psychiatry (2020) 87(9):e29–31. 10.1016/j.biopsych.2019.12.020 32122620

[127] Schmitt SE Pargeon K Frechette ES Hirsch LJ Dalmau J Friedman D Extreme delta brush: a unique EEG pattern in adults with anti-NMDA receptor encephalitis. Neurology (2012) 79(11):1094–100. 10.1212/WNL.0b013e3182698cd8 PMC3525298 22933737

[128] Endres D Leypoldt F Bechter K Autoimmune encephalitis as a differential diagnosis of schizophreniform psychosis: clinical symptomatology, pathophysiology, diagnostic approach, and therapeutic considerations. Eur Arch Psychiatry Clin Neurosci (2020). 10.1007/s00406-020-01113-2 PMC7474714 32166503

[129] Hufschmidt A Lücking CH Rauer S eds. Neurologie compact – Für Klinik und Praxis: Neuroradiologische Diagnostik und Praxis. 1st ed. Stuttgart: Thieme (2013).

[130] Peer M Prüss H Ben-Dayan I Paul F Arzy S Finke C Functional connectivity of large-scale brain networks in patients with anti-NMDA receptor encephalitis: an observational study. Lancet Psychiatry (2017) 4(10):768–74. 10.1016/S2215-0366(17)30330-9 28882707

[131] Titulaer MJ McCracken L Gabilondo I Armangue T Glaser C Iizuka T Treatment and prognostic factors for long-term outcome in patients with anti-N-Methyl-D-Aspartate (NMDA) receptor encephalitis: A cohort study. Lancet Neurol (2013) 12(2):157–65. 10.1016/S1474-4422(12)70310-1 PMC3563251 23290630

[132] van Tebartz Elst L Maier S Fangmeier T Endres D Mueller GT Nickel K Disturbed cingulate glutamate metabolism in adults with high-functioning autism spectrum disorder: evidence in support of the excitatory/inhibitory imbalance hypothesis. Mol Psychiatry (2014) 19(12):1314–25. 10.1038/mp.2014.62 25048006

[133] Cramer SP Modvig S Simonsen HJ Frederiksen JL Larsson HBW Permeability of the blood-brain barrier predicts conversion from optic neuritis to multiple sclerosis. Brain (2015) 138(Pt 9):2571–83. 10.1093/brain/awv203 PMC4547053 26187333

[134] Nickel K van Tebartz Elst L Holovics L Feige B Glauche V Fortenbacher T White Matter Abnormalities in the Corpus Callosum in Acute and Recovered Anorexia Nervosa Patients-A Diffusion Tensor Imaging Study. Front Psychiatry (2019) 10:490. 10.3389/fpsyt.2019.00490 31338044 PMC6628864

[135] Heine J Prüss H Bartsch T Ploner CJ Paul F Finke C Imaging of autoimmune encephalitis–Relevance for clinical practice and hippocampal function. Neuroscience (2015) 309:68–83. 10.1016/j.neuroscience.2015.05.037 26012492

[136] Hellwig S Domschke K Update on PET imaging biomarkers in the diagnosis of neuropsychiatric disorders. Curr Opin Neurol (2019) 32(4):539–47. 10.1097/WCO.0000000000000705 31083013

[137] Baumgartner A Rauer S Mader I Meyer PT Cerebral FDG-PET and MRI findings in autoimmune limbic encephalitis: correlation with autoantibody types. J Neurol (2013) 260(11):2744–53. 10.1007/s00415-013-7048-2 23900756

[138] Herken J Prüss H Red Flags: Clinical Signs for Identifying Autoimmune Encephalitis in Psychiatric Patients. Front Psychiatry (2017) 8:1–9. 10.3389/fpsyt.2017.00025 28261116 PMC5311041

[139] Konsman JP Reyes TM The lost cause of not being mechanistic enough? A perspective inspired by philosophy of science. Brain Behav Immun (2020) 84:1–3. 10.1016/j.bbi.2019.10.002 31610218

[140] Gable MS Sheriff H Dalmau J Tilley DH Glaser CA The frequency of autoimmune N-methyl-D-aspartate receptor encephalitis surpasses that of individual viral etiologies in young individuals enrolled in the California Encephalitis Project. Clin Infect Dis (2012) 54(7):899–904. 10.1093/cid/cir1038 22281844 PMC3297648

[141] Endres D Perlov E Stich O Rauer S Maier S Waldkircher Z Hypoglutamatergic state is associated with reduced cerebral glucose metabolism in anti-NMDA receptor encephalitis: A case report. BMC Psychiatry (2015) 15:1–8. 10.1186/s12888-015-0552-4 26231521 PMC4522073

[142] Mack A Pfeiffer C Schneider EM Bechter K Schizophrenia or Atypical Lupus Erythematosus with Predominant Psychiatric Manifestations over 25 Years: Case Analysis and Review. Front Psychiatry (2017) 8:1–9. 10.3389/fpsyt.2017.00131 28798699 PMC5530032

[143] Moises HW Wollschläger D Binder H Functional genomics indicate that schizophrenia may be an adult vascular-ischemic disorder. Transl Psychiatry (2015) 5(8):e616. 10.1038/tp.2015.103 PMC4564558 26261884

[144] Benros ME Eaton WW Mortensen PB The epidemiologic evidence linking autoimmune diseases and psychosis. Biol Psychiatry (2014) 75(4):300–6. 10.1016/j.biopsych.2013.09.023 PMC8797267 24199668

[145] Benros ME Mortensen PB Eaton WW Autoimmune diseases and infections as risk factors for schizophrenia. Ann N Y Acad Sci (2012) 1262:56–66. 10.1111/j.1749-6632.2012.06638.x 22823436

[146] Benros ME Waltoft BL Nordentoft M Ostergaard S Eaton WW Krogh J Autoimmune diseases and severe infections as risk factors for mood disorders: A nationwide study. JAMA Psychiatry (2013) 70(8):812–20. 10.1001/jamapsychiatry.2013.1111 23760347

[147] Nielsen PR Benros ME Mortensen PB Hospital contacts with infection and risk of schizophrenia: A population-based cohort study with linkage of Danish national registers. Schizophr Bull (2014) 40(6):1526–32. 10.1093/schbul/sbt200 PMC4193697 24379444

[148] Nielsen PR Kragstrup TW Deleuran BW Benros ME Infections as risk factor for autoimmune diseases - A nationwide study. J Autoimmun (2016) 74:176–81. 10.1016/j.jaut.2016.05.013 27267460

[149] Berk M Conus P Kapczinski F Andreazza AC Yücel M Wood S From neuroprogression to neuroprotection: implications for clinical care. Med J Aust (2010) 193(4 Suppl):S36–40. 20712560

[150] Kroken RA Sommer IE Steen VM Dieset I Johnsen E Constructing the Immune Signature of Schizophrenia for Clinical Use and Research; An Integrative Review Translating Descriptives Into Diagnostics. Front Psychiatry (2018) 9:753. 10.3389/fpsyt.2018.00753 30766494 PMC6365449

[151] Di Biase MA Zalesky A O’keefe G Laskaris L Baune B Weickert CS PET imaging of putative microglial activation in individuals at ultra-high risk for psychosis, recently diagnosed and chronically ill with schizophrenia. Transl Psychiatry (2017) 7(8):e1225. 10.1038/tp.2017.193 28850113 PMC5611755

[152] Cropley VL Pantelis C Using longitudinal imaging to map the ‘relapse signature’ of schizophrenia and other psychoses. Epidemiol Psychiatr Sci (2014) 23(3):219–25. 10.1017/S2045796014000341 PMC6998274 24849668

[153] Whitford TJ Wood SJ Yung A Cocchi L Berger G Shenton M Structural abnormalities in the cuneus associated with Herpes Simplex Virus (type 1) infection in people at ultra high risk of developing psychosis. Schizophr Res (2012) 135(1-3):175–80. 10.1016/j.schres.2011.11.003 PMC3405258 22244184

[154] Pollak TA Lennox BR Time for a change of practice: the real-world value of testing for neuronal autoantibodies in acute first-episode psychosis. BJPsych Open (2018) 4(4):262–4. 10.1192/bjo.2018.27 PMC6068314 30083377

